# Genome Sequence of the Cluster CD *Gordonia* Phage Widow

**DOI:** 10.1128/mra.00695-22

**Published:** 2022-09-06

**Authors:** Ben Curtis, Bayarjavkhlan Ganbaatar, Griffin Lawrence, Wyatt Oglesby, Jaylee Rice, Emelia Tremblay, Melissa Maginnis, Sally D. Molloy, Melody N. Neely

**Affiliations:** a Molecular and Biomedical Sciences, University of Maine, Orono, Maine, USA; b The Honors College, University of Maine, Orono, Maine, USA; DOE Joint Genome Institute

## Abstract

Widow is a novel cluster CD bacteriophage isolated from a soil sample using the bacterial host Gordonia terrae. The Widow genome is 43,656 bp in length and encodes 64 protein-coding genes and no tRNAs. The genome shares 52 to 92% gene content with other cluster CD members.

## ANNOUNCEMENT

**FIG 1:**
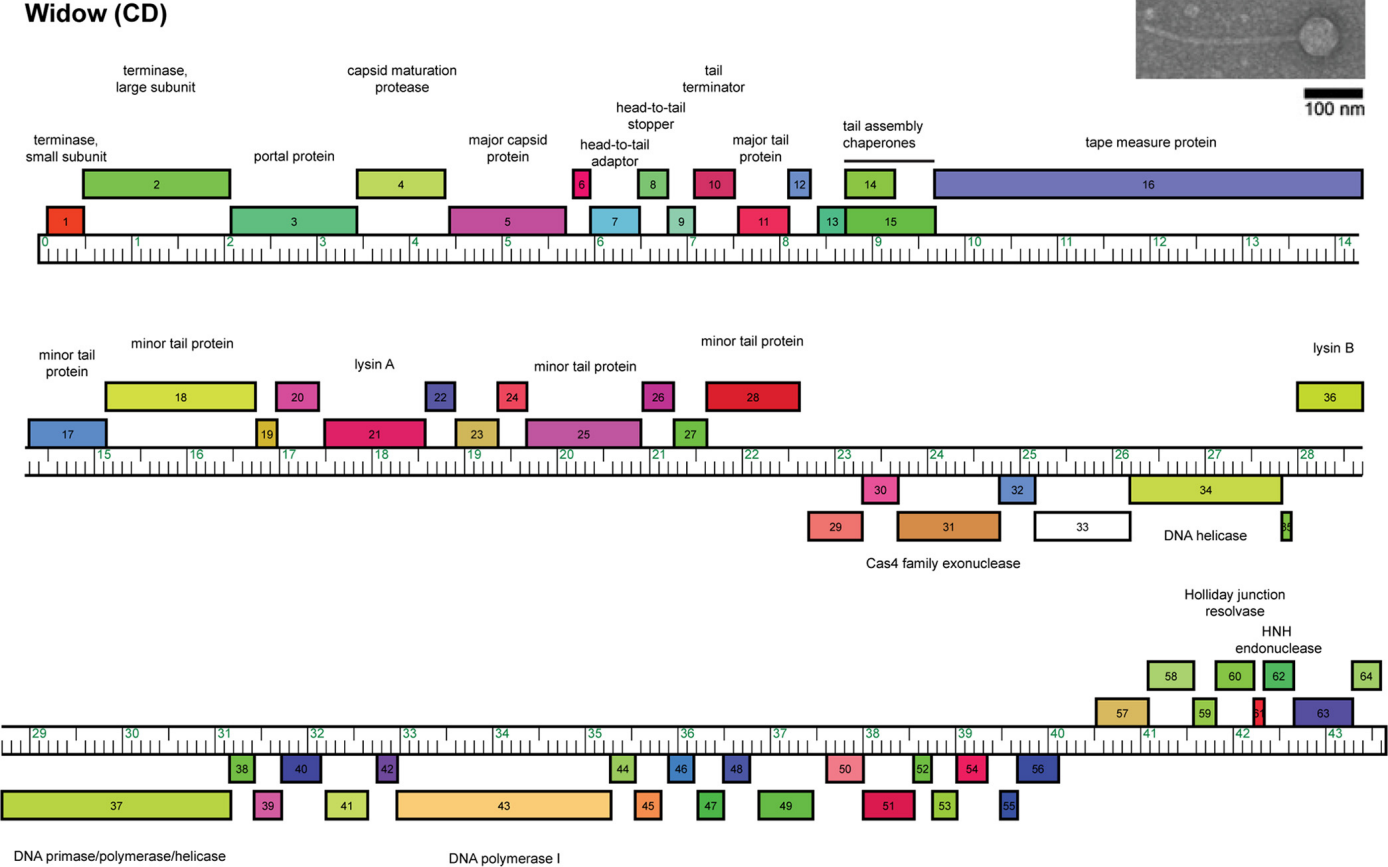
Genome map of *Gordonia* phage Widow. The genome coordinates are represented by the ruler in kilobase pair units. Each colored box above and below the ruler represents a gene transcribed in the forward or reverse direction, respectively. Genes were assigned to a phamily using Phamerator (9) in the Actino_draft database, and different phamilies are indicated by different colors. Genes that are orphams, i.e., phamilies with a single gene member, are colored white. An electron micrograph of Widow is shown in the inset, with a scale bar of 100 nm.

Actinobacteriophages, viruses that infect *Actinobacteria*, have diverse genomes that encode large numbers of uncharacterized genes with the potential to broaden our understanding of phage-host interactions and to develop antimicrobial treatments (1–5). The bacteriophage Widow was isolated from garden soil collected on 24 September 2021 in Mattapoiset, MA (41.658733°N, 70.805472°W) using the actinobacterial host Gordonia terrae 3612. An extract was prepared by incubating soil with peptone-yeast extract-calcium (PYCa) medium for 1 h at 30°C with shaking. The extract was centrifuged and filtered on a 0.22-μm filter before inoculating with *G. terrae* and incubating at 30°C for 2 days. The enriched extract was filtered, diluted, and plated in soft agar containing *G. terrae* onto PYCa agar. Widow produced clear plaques with a turbid halo, 2.5 mm in diameter, after 2 days of incubation at 30°C. Widow was purified by three rounds of plaque assays using standard methods (6). A high-titer lysate of Widow was examined by negative-stained transmission electron microscopy (6). Widow has a *Siphoviridae* morphology with a long, flexible, noncontractile tail, 274.5 ± 1.8 nm in length (mean ± standard error), and an icosahedral head that is 64.5 ± 0.9 nm in diameter (*n* = 6) (Fig. 1).

Using a phenol-chloroform extraction method, DNA was extracted from high-titer lysate and prepared for sequencing using the NEB Ultra II library kit (New England BioLabs, Ipswich, MA, USA) (7). Sequencing on an Illumina MiSeq platform resulted in 743,290 single-end 150-bp reads. Newbler v2.9 and Consed v29 were used to assemble and check for completeness, yielding a 43,656-bp genome with 67.6% GC content and 1,328-fold coverage. Genome ends were defined by single-stranded 11-bp 3′-extensions (TCCGCCGGTCT). Widow was assigned to cluster CD based on a shared gene content of greater than 35% with sequences in the Phamerator database Actino_Draft (5, 8, 9).

The Widow genome was autoannotated using DNA Master v5.23.6 (available from the Lawrence Lab website, http://cobamide2.bio.pitt.edu/) and PECAAN (https://blog.kbrinsgd.org/) with embedded programs Glimmer v3.02 and GeneMark v2.5 (10, 11). Translational starts were verified by identifying starts that included all GeneMark.hmm-predicted coding potential and were conserved across homologs according to Starterator (http://phages.wustl.edu/starterator/) and BLASTp (10, 12). Gene functions were predicted using BLAST, TMHMM, HHpred, and the Phamerator database Actino_Draft (9, 13, 14). All tools were run with default parameters unless otherwise specified. Aragorn v1.2.38 and tRNAscanSE detected no tRNA genes (15, 16). The genome encodes 64 protein-coding genes (Fig. 1). Beginning with the small and large terminase subunits (gp1 and gp2), the left arm encodes forward-transcribed structural and assembly genes (gp1 to gp28). The right arm of the genome encodes mainly reverse-oriented genes (gp29 to gp35 and gp37 to gp56), which are disrupted by a forward-transcribed lysin B gene (gp36) and followed by eight forward-transcribed genes (gp57 to gp64). The genome lacks genes required for lysogeny, such as an integrase and an immunity repressor (17).

Widow shares 51 to 92% gene content with the eight cluster CD members. The structural and assembly genes of the left arm are highly conserved across all the cluster CD phages, whereas the genes on the right arm are more varied. Widow encodes a single orpham (gp33), a gene phamily with a single member in the Actinobacteriophage database, with no known function. This is typical of cluster CD phages, which often encode orphams on the right arm of the genome.

Widow is available at GenBank under accession number ON456352 and Sequence Read Archive number SRX14816044.
